# Supplementary figures and images for: Oral aspirin for preventing colorectal adenoma recurrence: A systematic review and network meta-analysis of randomized controlled trials
Source: PLoS One. 2024 Mar 14;19(3):e0279784. doi: 10.1371/journal.pone.0279784 (PMC10939266; doi:10.1371/journal.pone.0279784)

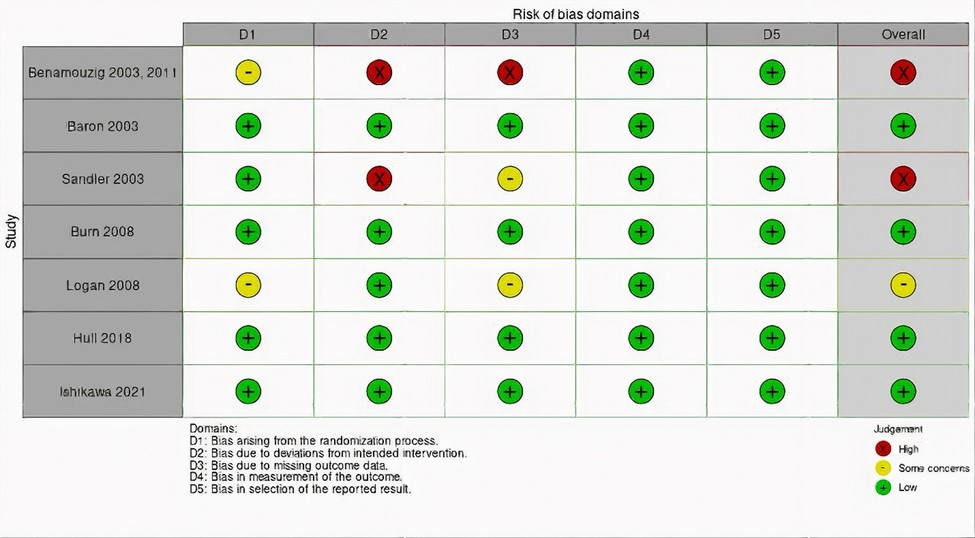

Supplement: S1 Fig — (TIF) [file pone.0279784.s001.tif]

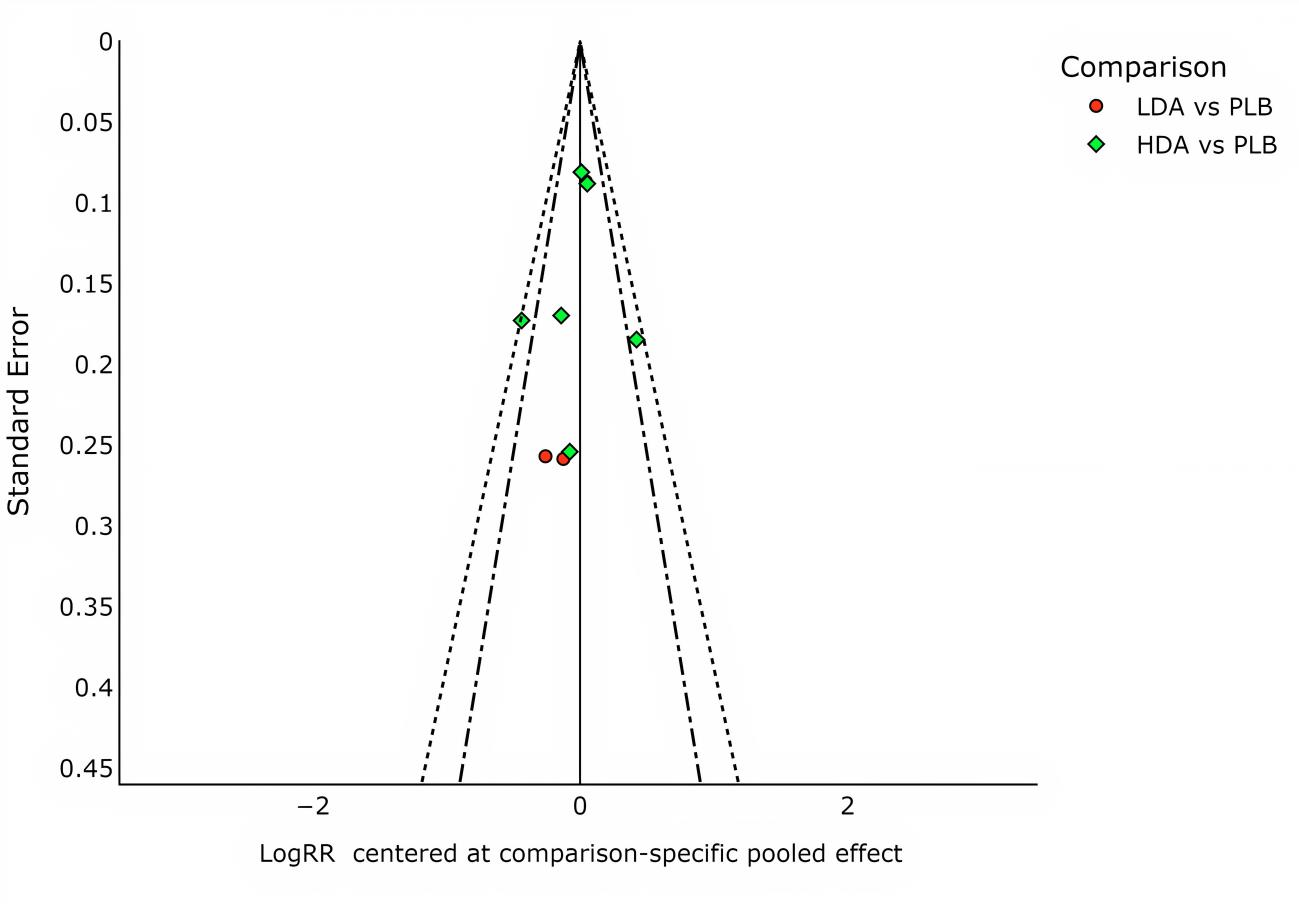

Supplement: S2 Fig — (TIF) [file pone.0279784.s002.tif]

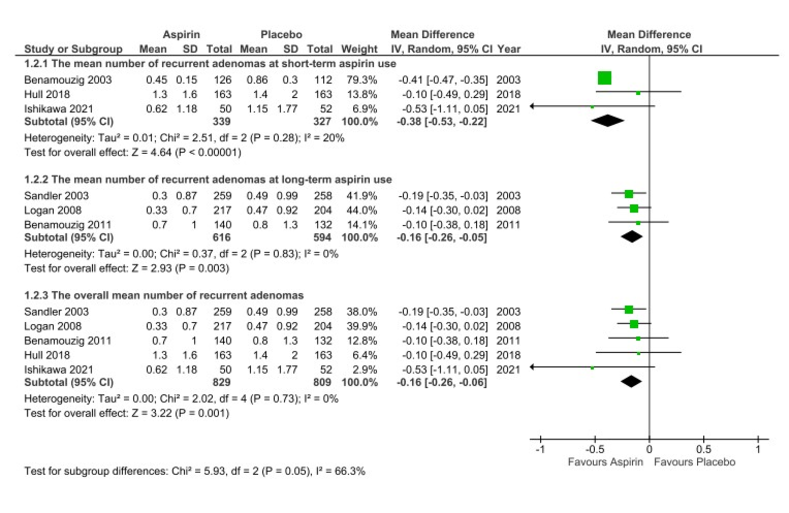

Supplement: S3 Fig — (TIF) [file pone.0279784.s003.tif]

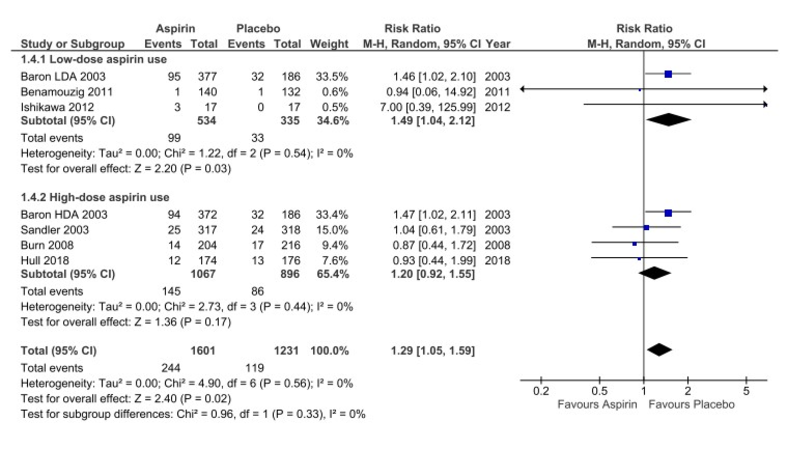

Supplement: S4 Fig — (TIF) [file pone.0279784.s004.tif]

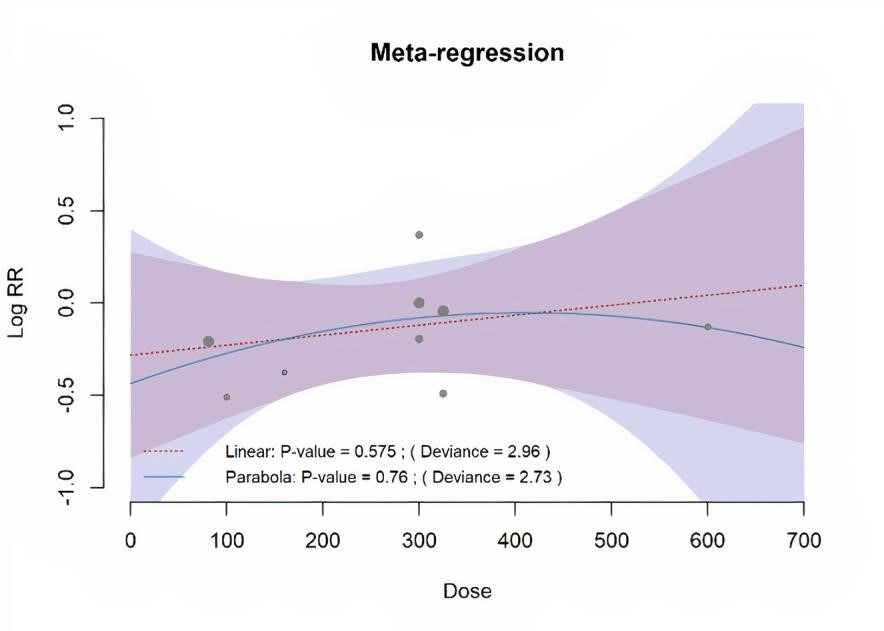

Supplement: S5 Fig — (TIF) [file pone.0279784.s005.tif]

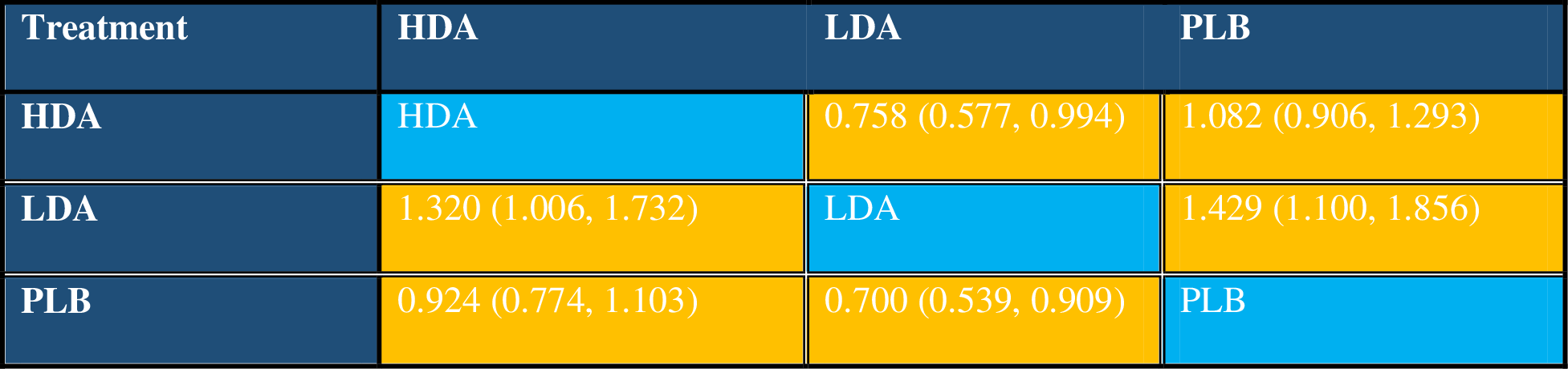

Supplement: S6 Fig — (TIF) [file pone.0279784.s006.tif]

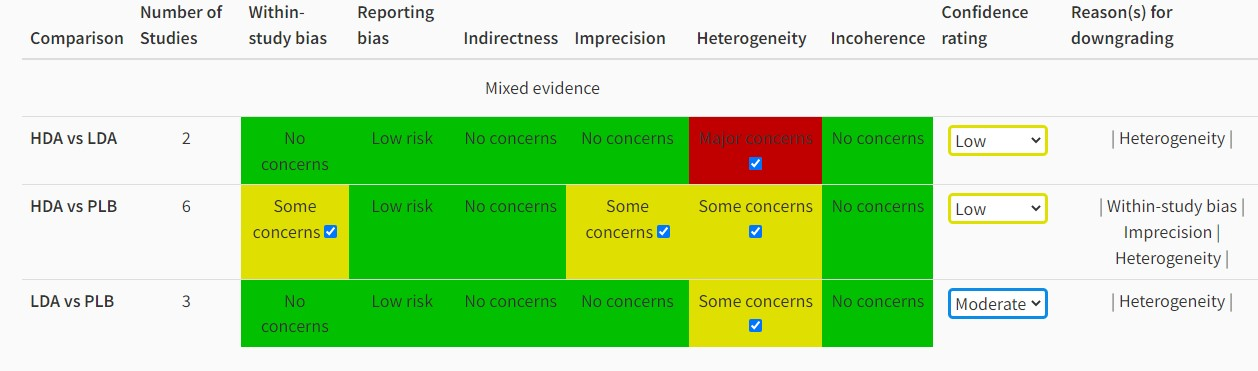

Supplement: S7 Fig — (TIF) [file pone.0279784.s007.tif]

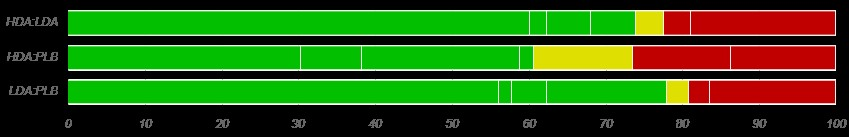

Supplement: S8 Fig — (TIF) [file pone.0279784.s008.tif]

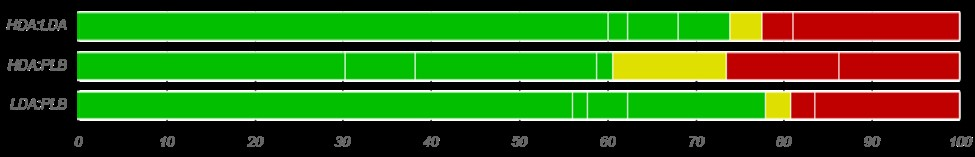

Supplement: S9 Fig — (TIF) [file pone.0279784.s009.tif]

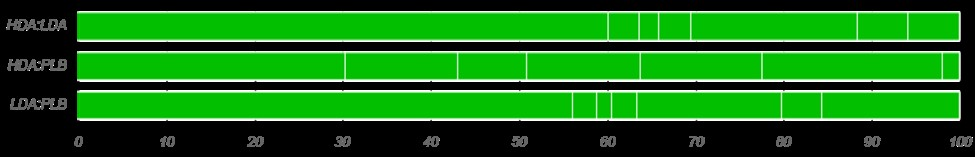

Supplement: S10 Fig — (TIF) [file pone.0279784.s010.tif]

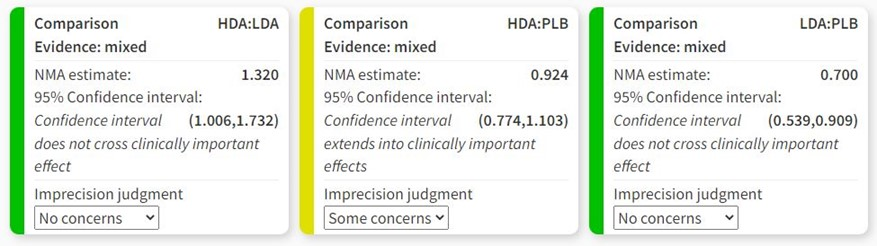

Supplement: S11 Fig — (TIF) [file pone.0279784.s011.tif]

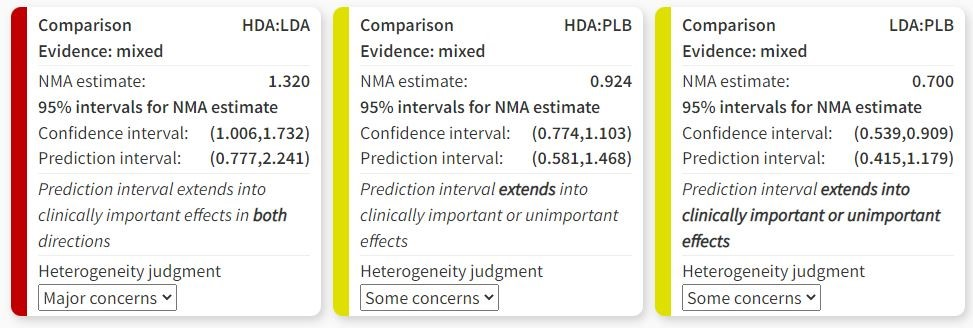

Supplement: S12 Fig — (TIF) [file pone.0279784.s012.tif]

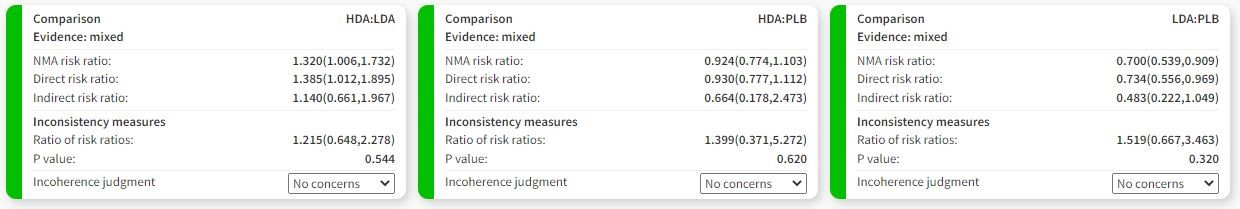

Supplement: S13 Fig — (TIF) [file pone.0279784.s013.tif]

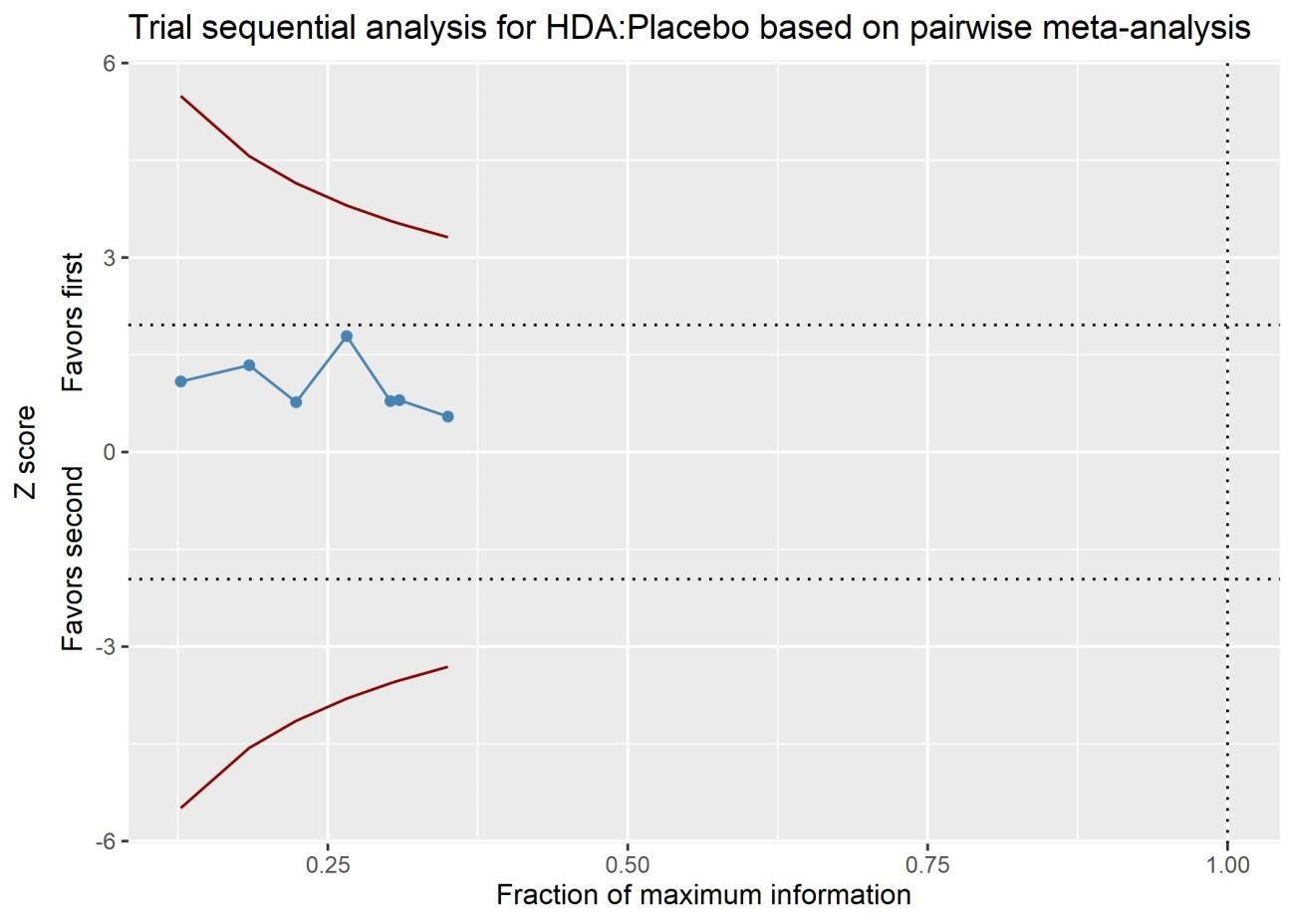

Supplement: S14 Fig — (TIF) [file pone.0279784.s014.tif]

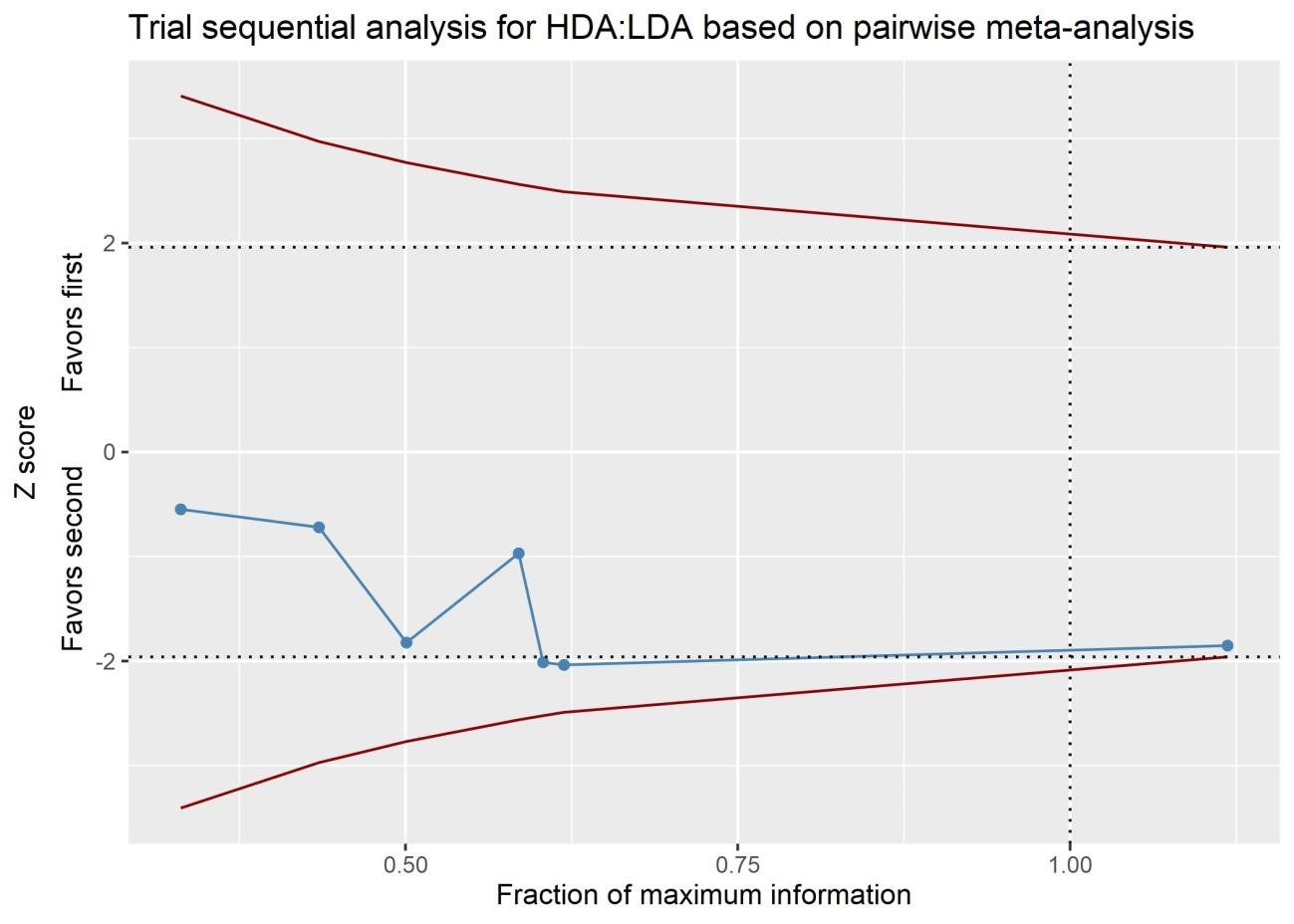

Supplement: S15 Fig — (TIF) [file pone.0279784.s015.tif]
