# Supplementary material for: Oral aspirin for preventing colorectal adenoma recurrence: A systematic review and network meta-analysis of randomized controlled trials
Source: PLoS One. 2024 Mar 14;19(3):e0279784. doi: 10.1371/journal.pone.0279784 (PMC10939266; doi:10.1371/journal.pone.0279784)
Supplement: Attachment — Submitted filename: Response to Reviewers.pdf [file pone.0279784.s018.pdf]

Response to reviewer

Chiehfeng Chen
Department of Public Health, College of Medicine, Taipei Medical University
250 Wuxing Street, Xinyi District, Taipei, Taiwan (R.O.C)

Septerber 10th, 2023

Dear Reviewers,

First of all, we would like to thank you for your consideration and your valuable comments on our manuscript. Thanks to your comments, we realize that our manuscript still has some shortcomings. We have tried to perfect our manuscript as best we could based on your comments.

Comment #1: Please review your reference list to ensure that it is complete and correct. If you have cited papers that have been retracted, please include the rationale for doing so in the manuscript text, or remove these references and replace them with relevant current references. Any changes to the reference list should be mentioned in the rebuttal letter that accompanies your revised manuscript. If you need to cite a retracted article, indicate the article's retracted status in the References list and also include a citation and full reference for the retraction notice.

Authors: We thoroughly reviewed the reference lists multiple times according to your comment and found that they are totally accurate. Up to now, none of these references have been retracted from online database. Therefore, we kept the reference list remain unchanged.

Additional Editor Comments: The manuscript needs to be corrected for English language and poor grammatical construction. Once a revised manuscript is submitted that does not include grammatical errors, I will send it out for re-review.

Authors: We found several problems with poor grammartical structures and English language, so we rewrote some paragraphs in the 'Discussion' section. Any modifications were highlighted in 'Revised Manuscript with Track Changes'.

One more time, thank you for your consideration of this manuscript. We hope our manuscript to receive good responses from you and then to be accepted by the journal.

Sincerely,

Chiehfeng Chen MD. MPH. PhD.

Department of Public Health, School of Medicine, College of Medicine, and Cochrane Taiwan, Taipei Medical University, 250 Wuxing St., Taipei 11031, Taiwan

Email address: clifchen@tmu.edu.tw

Phone number: 886-2-2930-7930 ext. 8105
