# Supplementary material for: Oral aspirin for preventing colorectal adenoma recurrence: A systematic review and network meta-analysis of randomized controlled trials
Source: PLoS One. 2024 Mar 14;19(3):e0279784. doi: 10.1371/journal.pone.0279784 (PMC10939266; doi:10.1371/journal.pone.0279784)
Supplement: Attachment — Submitted filename: Response to Reviewers.docx [file pone.0279784.s017.docx]

**Response to reviewer**

Chiehfeng Chen
Department of Public Health, College of Medicine, Taipei Medical University
250 Wuxing Street, Xinyi District, Taipei, Taiwan (R.O.C)

April 15, 2023

Dear Reviewers,

First of all, we would like to thank you for your consideration and your valuable comments on our manuscript. Thanks to your comments, we realize that our manuscript still has some shortcomings. We have tried to perfect our manuscript as best we could based on your comments.

Comment #1: The introduction of the article is unnecessarily long and filled with excessive jargon which is not really required. For instance, the details regarding the pathophysiology is not needed in the introduction and can be removed. The authors should cut down on the text of the introduction and make it as comprehensive and readable as possible. Consider the following sequence for a better introduction - first mention the known details with relevant statistics and what the current guidelines state about oral aspirin for colorectal adenoma recurrence, then mention the conflicts in literature and what is the basis for investigating this and finally develop strongly on the rationale of the meta-analysis and mention the main objective of the study.

Authors: We omitted unnecessary parts about the pathogenesis of colorectal adenomas and the mechanism of action of aspirin. In addition, we included some relevant statistics and guidelines on the use of oral aspirin for colorectal adenomas prevention by Cancer Council Australia.

Comment #2: Consider including a subheading for conclusion and mention the conclusive statements there.

Authors: We included a subheading for conclusion and mention the conclusive statements there.

Comment #3 and #6: The discussion does not elaborate upon the future directions for oral aspirin use for preventing colorectal adenoma recurrences. Kindly add a paragraph for it; The discussion lacks details about clinical implications of this meta-analysis. Kindly add a paragraph for it.

Authors: We added a paragraph indicating the futute use of oral aspirin to prevent colorectal adenoma recurrence, as well as the clinical implications for this meta-analysis in the same paragraph as follows:

“From all of the above analyses, it could be concluded that LDA was more effective than placebo in averting colorectal adenoma recurrence, especially in the long-term use. But apparently, there was insufficient proof to confirm whether LDA or LDA was more protective. Besides, LDA was also highly efficacious in cardiovascular diseases prevention. We believe that LDA (< 300 mg per day) should be applied clinically to preclude colorectal adenoma recurrence as well as several other conditions. But apparently, there was insufficient proof to confirm whether LDA or HDA was more protective in colorectal adenoma recurrence based on trial sequential analysisa and meta-regression. Thus, more clinical trials need to be conducted to evaluate the effectiveness of LDA compared with HDA. However, this may not be feasible, given the large number of participants included in this analysis, meaning that a large number of studies are more needed to make a significant change.”

Comment #4: The authors state in the first paragraph of discussion, 'its efficacy was more statistically significant for long-term use than for short-term use.' Please rephrase this sentence. Just saying more statistically significant does not indicate any direction of the results.

Authors: we rephrased that sentence as your request: “and long-term use of LDA was more protective than short-term use.”

Comment #5: The authors state 'These results are not easy to explain' in the discussion while mentioning the large trial by Baron et al. This is a very important study and given the fact that it is well-designed and robustly conducted, removing this study from the analysis should have a solid reason which must be stated in the text. Please also discuss why the risk of severe adverse events was significantly higher in the aspirin group in this study. While it may be hard to state definitive reasons, the authors can provide suggestive reasons as to why this was the case in this large trial.

Authors: We tried to come up with some possible reasons to explain the high incidence of adverse events performed by Baron 2003 study. (follow-up period was quite long, the reasons for hospitalization were not clearly indicated so that it was impossible to make sure that those events were induced by aspirin or other medical conditions acquired during follow-up.

One more time, thank you for your consideration of this manuscript. We hope our manuscript to receive good responses from you and then to be accepted by the journal.

Sincerely,

Chiehfeng Chen MD. MPH. PhD.

Department of Public Health, School of Medicine, College of Medicine, and Cochrane Taiwan, Taipei Medical University, 250 Wuxing St., Taipei 11031, Taiwan

Email address: clifchen@tmu.edu.tw

Phone number: 886-2-2930-7930 ext. 8105
